# Electroconvulsive therapy in the psychiatric depatement of the Mahdia EPS over two years

**DOI:** 10.1192/j.eurpsy.2021.1312

**Published:** 2021-08-13

**Authors:** S. Brahim, W. Bouali, M. Henia, A. Abid, M. Chabbouh, L. Zarrouk

**Affiliations:** 1 Psychiatry, University Hospital of Mahdia, Tunisia., chebba, Tunisia; 2 Department Of Psychiatry, University Hospital Of Mahdia, Tunisia., Psychiatry, Mahdia, Tunisia; 3 Anesthesia, University Hospital of Mahdia, Tunisia., mahdia, Tunisia; 4 Psychiatry, University Hospital of Mahdia, Tunisia., Mahdia, Tunisia

**Keywords:** electoconvulsive therapy, Treatment, Suicide, bipolar disorder

## Abstract

**Introduction:**

The electroconvulsive therapy is an ancient therapeutic technique used in the traitment of certain psychiatric diseases.

**Objectives:**

discuss the technical aspects, indications, therapeutic response and tolerance of ECT

**Methods:**

This was a descriptive retrospective study that interested all patients who were hospitalized in the psychiatric department of the Mahdia University Hospital in 2017 and 2018 and were benefited from ECT sessions

**Results:**

The number of patients who received ECT was 34, representing 4.33% of patients, 25 men and 9 women with an average age of 39, the number of ECT sessions was 785. The major diagnosis was bipolar disorder in 47,1% of patients, followed by schizophrenia in 35,3% and major depressive disorder in 14,7 %. Resistance to treatment and major suicidal risk were the main indications. All sessions were performed in a bilateral temporal mode. the initial energy delivered varied between 50 and 101 millicoulombs. The duration of the crise obtained was predominantly between 21 and 30 seconds. The average number of sessions during the attack phase was 13.88, whereas it was 2.5 sessions during the consolidation phase. The mean scores of the psychometric evaluations showed a marked improvement, especially in the mania scores (65.89%) and the beck depression inventory (63.55%). Only four incidents were reported in all patients. Only five patients (14,7%) had side effects and the most marked effect was anterograde amnesia.

**Conclusions:**

Mental health programs in Tunisia should promote the generalization of this method throughout the Tunisian territory, given the efficacy demonstrated in mood disorder, several psychoses and other psychiatric pathologie.

